# Deep learning-based uncertainty quantification for quality assurance in hepatobiliary imaging-based techniques

**DOI:** 10.18632/oncotarget.28709

**Published:** 2025-04-04

**Authors:** Yashbir Singh, Jesper B. Andersen, Quincy Hathaway, Sudhakar K. Venkatesh, Gregory J. Gores, Bradley Erickson

**Keywords:** deep learning, uncertainty quantification, radiology, hepatobiliary imaging

## Abstract

Recent advances in deep learning models have transformed medical imaging analysis, particularly in radiology. This editorial outlines how uncertainty quantification through embedding-based approaches enhances diagnostic accuracy and reliability in hepatobiliary imaging, with a specific focus on oncological conditions and early detection of precancerous lesions. We explore modern architectures like the Anisotropic Hybrid Network (AHUNet), which leverages both 2D imaging and 3D volumetric data through innovative convolutional approaches. We consider the implications for quality assurance in radiological practice and discuss recent clinical applications.

## INTRODUCTION

Artificial intelligence applying deep learning models has revolutionized medical imaging analysis by enabling automated lesion detection, enhancing organ segmentation accuracy, and reducing inter-observer variability. This may be of particular significance in hepatobiliary radiology, where precise interpretation and quality assurance are paramount. This is due to the complex anatomical relationships between structures within the liver and biliary system, often in a background of fibrotic scarring or liver cirrhosis that can directly impact surgical planning and treatment approaches. Additionally, there is a frequent need to differentiate between benign and malignant lesions [1]. The integration of deep learning-based uncertainty quantification in medical imaging analysis addresses several key challenges, including variable image quality, anatomical variations, and the presence of artifacts (including motion artifacts from patient movement, beam hardening in computed tomography (CT), metal implant artifacts creating streaks, partial volume effects, noise from low radiation doses, reconstruction algorithm limitations, scanner calibration issues, phase wrap and chemical shift artifacts in magnetic resonance imaging (MRI)) that have historically complicated radiological assessments, and inter-radiologist variability due to differing levels of experience [2]. These models effectively and automatically analyse complex imaging patterns while providing quantifiable confidence measures as precision for their predictions [3]. These advancements are valuable given the intricate nature of liver pathologies and the critical importance of early detection in conditions like hepatocellular carcinoma (HCC) and intrahepatic cholangiocarcinoma (iCCA). It is crucial to correctly identify various liver cancers as treatment strategies and prognosis vary. HCCs are usually treated with locoregional methods (embolization, thermal ablation, radiation etc) targeted kinase inhibitors or immunotherapy, whereas iCCA is usually treated with systemic chemotherapy sometimes combined with radiotherapy or immunotherapy.

### Deep learning architectures

#### Uncertainty quantification for hepatobiliary disease

**Figure 1:**
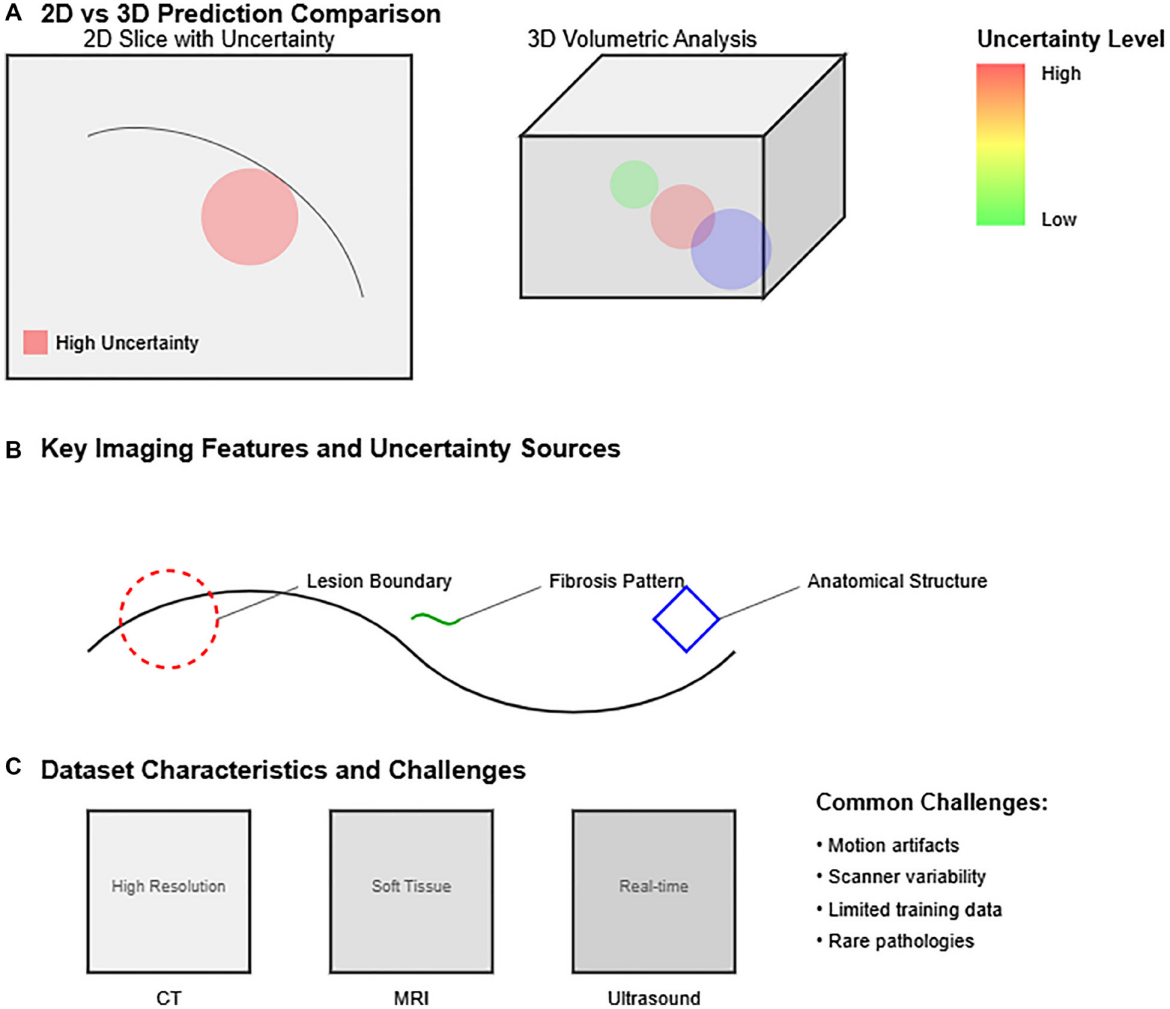
Uncertainty quantification in deep learning-based hepatobiliary imaging analysis. (**A**) 2D vs. 3D Prediction comparison: Visualization of uncertainty quantification approaches in hepatobiliary imaging. The 2D analysis (left) shows a liver slice with uncertainty overlay, where red regions indicate areas of high uncertainty in lesion detection and boundary determination. The intensity of the color corresponds to the model’s confidence level, as demonstrated in the AHUNet framework (Lambert et al., 2023). The 3D volumetric reconstruction (right) illustrates how uncertainty propagates across multiple slices, particularly important for analyzing complex anatomical relationships and detecting small lesions (Dice score 0.57 for focal lesions vs. 0.94 for total liver volume). (**B**) Key imaging features and uncertainty sources: Demonstration of critical features that contribute to uncertainty in predictions. Lesion boundaries (red dashed lines) often present higher uncertainty due to partial volume effects and unclear margins, especially in the context of background liver cirrhosis. Fibrosis patterns (green) represent areas where tissue heterogeneity can affect model confidence, particularly relevant in T1ρ mapping applications (Huang et al., 2023). Anatomical structures (blue) show how structural relationships influence uncertainty quantification, especially in cases with variable image quality or motion artifacts. (**C**) Dataset characteristics and challenges: Representation of different imaging modalities (CT, MRI, ultrasound) and their specific challenges in uncertainty quantification. CT images provide high spatial resolution but face challenges with radiation dose optimization. MRI excels in soft tissue contrast but is susceptible to motion artifacts and longer acquisition times. Ultrasound offers real-time imaging but presents challenges in standardization and operator dependency, as demonstrated in recent fatty liver content assessment studies (Del Corso et al., 2024). Common challenges across modalities include scanner variability, limited training data for rare pathologies, and motion artifacts, which directly impact the reliability of uncertainty estimates. The color gradient legend indicates uncertainty levels, with red representing high uncertainty regions requiring additional radiologist attention, and green indicating areas of high confidence in model predictions. This quantification framework aligns with clinical implementation guidelines, where uncertainty scores can trigger additional review or modified follow-up protocols in both screening and diagnostic contexts. This visualization illustrates the comprehensive approach to uncertainty quantification described in the paper, emphasizing the integration of both aleatoric uncertainty (inherent in imaging data) and epistemic uncertainty (model-related), while highlighting the practical challenges in clinical implementation.

Modern deep learning architectures leverage sophisticated embedding techniques to capture subtle variations in imaging characteristics [4, 5]. These embeddings serve as high-dimensional representations of radiological features, enabling more nuanced analysis than traditional methods [6]. The uncertainty quantification framework operates on multiple levels, assessing both aleatoric uncertainty (inherent noise in the data) and epistemic uncertainty (model uncertainty) [7–9]. Lambert et al. [10] utilized an Anisotropic Hybrid Network (AHUNet) to handle the inherent anisotropy of medical images (i.e., anisotropy referring to the variation in cell types and density of an organ that ultimately produces a spectrum of imaging features) [10]. This architecture performed well in the segmentation of the total liver volume (Dice 0.94) but was more imprecise in detecting specific focal lesions (Dice: 0.57). Further, the authors showed that the algorithm was more “uncertain” when dealing with smaller lesions and in a multi-lesion setting. AHUNet frameworks can introduce lesion-level confidence scoring, calculated from aggregated voxel-wise tumor probabilities, proving particularly effective in discriminating between true and false positive lesions [10] (Figure 1).

Building upon these initial approaches to uncertainty quantification in medical imaging, subsequent research has explored alternative probabilistic architectures to further improve prediction reliability. A 2024 study by Del Corso et al. [11] evaluated three probabilistic convolutional architectures for assessing the fatty liver content (FLC) from ultrasound imaging: a classical Convolutional Neural Network (CNN), Monte Carlo Dropout CNN, and Bayesian CNN. Testing these prediction algorithms on 186 patients with contrast-enhanced ultrasound images, they found Monte Carlo Dropout achieved the best regression performance (RMSE 5.35%) while maintaining reasonable uncertainty estimates (CoV 0.32). The Bayesian CNN improved classification accuracy from 86.1% to 91.7% by incorporating uncertainty scores, though at the cost of predicting more cases as uncertain (22.6% vs. 12.9% for Monte Carlo Dropout). While computationally more intensive, their work demonstrated that adding uncertainty quantification, provides valuable reliability metrics for clinical decision support, particularly in cases with poor image quality or atypical presentations [11]. Notably, while these CNN algorithms excel in 2D image analysis, AHUNet’s hybrid approach offers superior performance in volumetric analysis through its ability to preserve spatial relationships across image slices. However, this performance comes at the cost of increased computational complexity and training data requirements, even beyond the typical large datasets needed for standard deep learning models. These computational requirements are due to the need to learn complex 3D spatial relationships across several image slices. The AHUNet’s improved handling of anisotropic features suggests a promising direction for future architectural developments, particularly in multi-modal imaging integration. A fundamental consideration for advancing these approaches is the challenge of acquiring and curating high-quality training datasets that are sufficiently large and diverse (e.g., multi-institutional) to enable robust model development, while ensuring proper validation across different patient populations and imaging protocols (Table 1). To push the field forward, we propose several key directions for future research:

1. Development of adaptive architectures that can dynamically switch between 2D and 3D processing based on input characteristics
2. Integration of multi-scale uncertainty quantification to better handle varying lesion sizes
3. Exploration of self-supervised learning approaches to address the limited availability of labeled medical imaging data
4. Investigation of lightweight uncertainty quantification methods to reduce computational overhead while maintaining accuracy

**Table 1:** Evolution of uncertainty quantification methods in hepatobiliary imaging from traditional to AI-based approaches

Time Period	Method/Architecture	Key features	Performance metrics	Clinical applications	Limitations
Traditional Methods (Pre-2020)					
Pre-2015	Expert Consensus	- Visual assessment - Inter-reader agreement - Standardized reporting systems	- Inter-observer variability - Subjective confidence levels	- Lesion characterization - Treatment planning	- High variability - Time-consuming - Limited reproducibility
2015–2019	Statistical Analysis	- ROI-based measurements - Texture analysis - Radiomics features	- Sensitivity/Specificity - Confidence intervals	- Tumor detection - Fibrosis staging	- Limited automation - Requires manual input
Modern AI-Based Methods (2020-Present)					
2020–2022	Classical CNN	- 2D image analysis - Basic uncertainty estimates	- Classification accuracy: 86.1% - Uncertainty rate: 12.9%	- Fatty liver assessment - Basic lesion detection	- Limited to 2D analysis - No spatial context preservation
2023	AHUNet	- Hybrid 2D/3D processing - Anisotropic feature handling - Lesion-level confidence scoring	- Liver volume Dice: 0.94 - Focal lesion Dice: 0.57	- Tumor segmentation - Volume estimation - Multi-lesion detection	- High computational cost - Large training data requirements
2023–2024	Monte Carlo Dropout CNN	- Probabilistic predictions - Dropout-based uncertainty	- RMSE: 5.35% - CoV: 0.32 - Better regression performance	- Fatty liver content assessment - Image quality assessment	- Computationally intensive - Limited to specific applications
2024	Bayesian CNN	- Probabilistic framework - Comprehensive uncertainty modeling	- Classification accuracy: 91.7% - Uncertainty rate: 22.6%	- Complex lesion characterization - Quality assurance	- Higher uncertainty predictions - Complex implementation
2024	UP-Net	- Physics-driven approach - Dual-module architecture - GAN-based artifact suppression	- Processing time: 79 ms/slice - Improved quantification accuracy	- Fat fraction quantification - R2^*^ mapping - Real-time processing	- Requires physics modeling - Complex architecture

The table presents a chronological progression of uncertainty quantification methods in hepatobiliary imaging, spanning from traditional expert-based approaches to modern artificial intelligence techniques (2015–2024). Methods are categorized by time period and architectural approach, with detailed descriptions of key features, quantitative performance metrics, clinical applications, and inherent limitations. Traditional methods (Pre-2020) demonstrate the transition from subjective expert consensus to statistical analysis approaches. Modern AI-based methods (2020-Present) show the development from classical convolutional neural networks to sophisticated architectures like AHUNet, Monte Carlo Dropout CNN, Bayesian CNN, and physics-driven networks (UP-Net). Performance metrics highlight improvements in accuracy (from basic inter-observer agreement to 91.7% classification accuracy) and processing efficiency (achieving 79 ms/slice processing times). The progression illustrates the field’s evolution toward quantitative, automated, and clinically integrated uncertainty assessment methods, while acknowledging computational and implementation challenges. Data compiled from referenced studies including Del Corso et al. (2024), Lambert et al. (2023), and others cited in the manuscript.

These advancements, by providing more reliable and efficient diagnostic tools, may significantly impact clinical practice by addressing the ever-growing clinical demand and work pressure, while maintaining interpretability and clinical relevance.

#### Uncertainty quantification for hepatobiliary image processing

Recent studies have demonstrated the practical implementation of uncertainty quantification across various hepatobiliary imaging applications. These investigations highlight the technical capacity of using deep learning algorithms to improve image reconstruction and calculation of uncertainty. Huang et al. [12] introduced a learning-based framework for liver T1ρ mapping (a specialized MRI technique that measures the spin-lattice relaxation time in the rotating frame, which is useful for detecting early biochemical changes in the liver parenchyma) with integrated uncertainty estimation. This approach employed a probabilistic neural network to refine coarse T1ρ maps from reduced T1ρ-weighted images while simultaneously generating uncertainty measures. In a study of 51 patients with varying stages of liver fibrosis, the system achieved less than 3% relative mapping error and effectively identified unreliable regions. Region of interest (ROI) refinement and quantification accuracy improved through uncertainty-weighted training and scan times decreased from ten to six seconds while maintaining accuracy, showing promise for clinical applications requiring rapid T1ρ quantification [12]. However, the performance of this model has yet to be validated on an independent external dataset, which would be crucial for establishing its generalizability across different clinical settings and patient populations.

**Figure 2:**
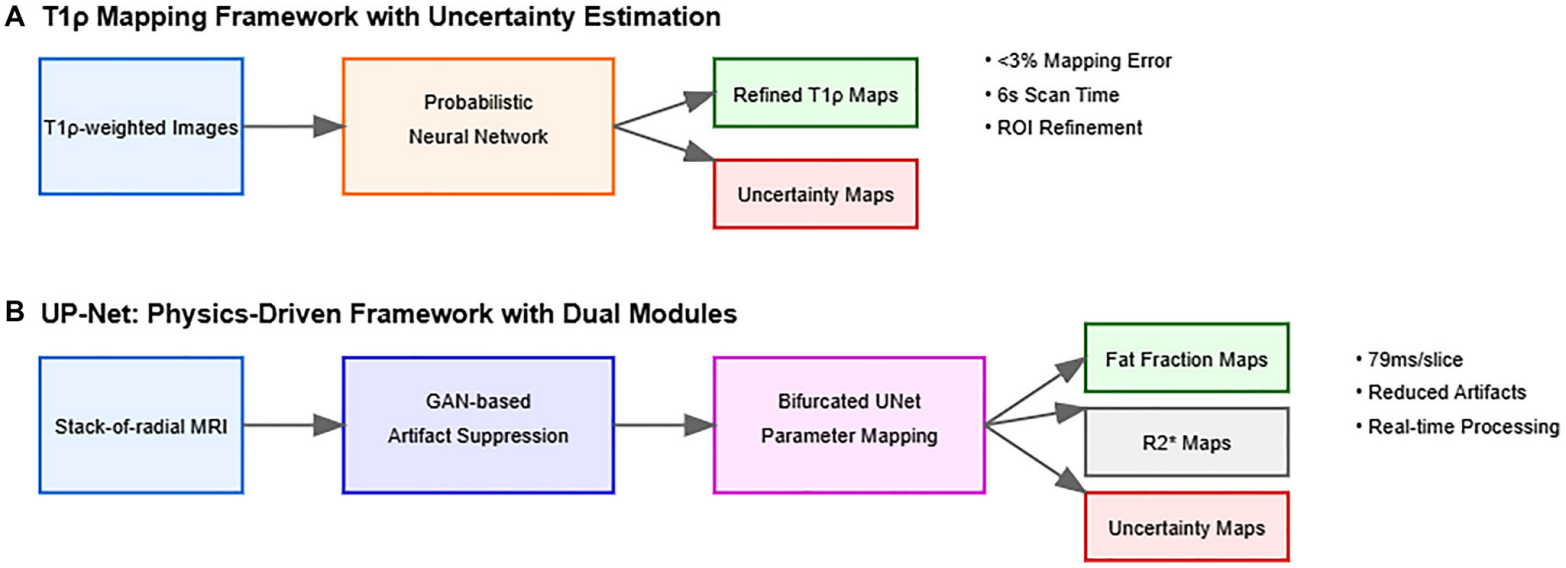
Architectural frameworks for uncertainty quantification in hepatobiliary image processing. (**A**) T1ρ mapping framework with integrated uncertainty estimation. The probabilistic neural network processes T1ρ-weighted images to simultaneously generate refined T1ρ maps and uncertainty estimates. This framework achieved <3% relative mapping error while reducing scan times from 10 to 6 seconds (Huang et al., 2023). The uncertainty-weighted training enables effective ROI refinement and identification of unreliable regions. (**B**) UP-Net dual-module physics-driven architecture. The first module employs GAN-based artifact suppression to reduce radial streak artifacts in stack-of-radial MRI data. The second module uses a bifurcated UNet structure for parameter mapping, generating quantitative maps (fat fraction and R2^*^) along with uncertainty estimates. This framework dramatically improved processing efficiency (79 ms/slice vs. 3.2 min/slice) while maintaining accuracy through uncertainty-weighted training (Shih et al., 2023). Color coding indicates different processing stages: input data (blue), intermediate processing modules (orange/purple), and output maps (green for quantitative maps, red for uncertainty estimates). Arrows show the data flow through each framework, illustrating how uncertainty quantification is integrated into the processing pipeline. Both architectures demonstrate the evolution toward real-time processing capabilities while maintaining robust uncertainty estimation for clinical applications.

Shih et al. [13] developed an uncertainty-aware physics-driven deep learning network (UP-Net) for proton-density fat fraction and R2^*^ quantification self-gated free-breathing stack-of-radial MRI. Their framework used a two-module approach: an artifact suppression module employing generative adversarial networks (GAN) to reduce radial streak artifacts and a parameter mapping module with a bifurcated UNet structure to generate quantitative maps and uncertainty estimates simultaneously. The uncertainty maps effectively identified unreliable regions and improved quantification accuracy through uncertainty-weighted training, decreasing slice reconstruction time from 3.2 min/slice (using standard approaches) to 79 ms/slice. Their approach demonstrates how integrating uncertainty estimation can enhance both the accuracy and reliability of quantitative MRI analysis [13]. These advancements represent significant progress in applying uncertainty quantification to hepatobiliary imaging, with three key developments emerging: First, the dramatic reduction in processing times while still maintaining or improving accuracy suggests these methods are becoming clinically viable and reliable. Second, the integration of physics-driven approaches with deep learning demonstrates a mature understanding of both computational and domain-specific challenges (such as MRI physics, anatomical variability in the hepatobiliary system, and clinical workflow requirements). Finally, the consistent improvement in accuracy across different imaging modalities and applications indicates the robustness of uncertainty quantification as a methodological framework (Figure 2). Together, these developments suggest we are approaching a paradigm shift in quantitative medical imaging, where uncertainty awareness becomes an integral part of clinical image analysis.

### Quality assurance and future implications

Quality assurance in hepatobiliary imaging has become increasingly crucial as imaging protocols and modalities evolve [9]. Deep learning models can automatically verify protocol adherence, image quality, and technical parameters across large datasets. The power of embedding-based approaches lies in their ability to learn from diverse datasets while maintaining robustness to variations in imaging parameters and patient characteristics. One significant advantage is the ability to identify out-of-distribution cases - situations where imaging characteristics deviate significantly from the training distribution. These deviations can stem from multiple sources of variability: inter-operator differences in image acquisition techniques, variations between imaging equipment and protocols across different institutions, patient-specific anatomical variations, and differences in technical parameters. By detecting these variations, the models can identify cases that fall outside their reliable operating range. This capability is crucial for flagging unusual presentations or potential artifacts requiring special attention.

### Clinical implementation and practical considerations

#### Integration with clinical workflows

The adoption of uncertainty quantification methods in hepatobiliary imaging demands careful alignment with existing radiological workflows [6, 9]. Radiology departments should develop standardized reporting templates that incorporate uncertainty metrics alongside traditional imaging findings. These templates should clearly communicate both the degree of confidence in specific findings and any areas requiring additional attention. For instance, in cases of HCC screening, uncertainty scores can be mapped to LI-RADS categories, providing an additional layer of confidence assessment [8]. The integration process should maintain efficiency while ensuring that uncertainty information enhances, rather than complicates, clinical decision-making [14].

#### Interpretation guidelines and training requirements

Radiologists require specific training to effectively interpret and utilize uncertainty quantification metrics [2]. Understanding the distinction between aleatoric uncertainty (inherent in the imaging data) and epistemic uncertainty (model-related) is crucial for proper clinical application [7]. Training programs should focus on practical case-based scenarios, demonstrating how uncertainty measures correlate with clinical outcomes, as shown in recent T1ρ mapping studies [12]. Common pitfalls include over-reliance on uncertainty scores without considering clinical context and misinterpretation of uncertainty thresholds in different clinical scenarios. Departments could establish regular quality assurance meetings to review cases where uncertainty quantification significantly influenced clinical decisions [9]. This quality assurance framework could be extended to create regional imaging hubs where larger centers provide support to smaller hospitals, enabling them to access advanced uncertainty quantification expertise and validation services, particularly beneficial for facilities with limited specialized staff.

#### Decision support framework

The application of uncertainty quantification varies across different clinical contexts, as demonstrated by recent developments in AHUNet frameworks [10]. In screening settings, higher uncertainty thresholds may trigger additional imaging or shortened follow-up intervals. For diagnostic imaging, uncertainty scores can help prioritize cases for subspecialist consultation [3]. These measures are particularly valuable in multidisciplinary tumor boards, where quantifiable confidence levels can inform treatment planning decisions [8]. The effectiveness of this approach has been recently shown [13], where uncertainty measures successfully identified unreliable regions requiring additional attention. Specifically, the study demonstrated that regions flagged with high uncertainty scores correlated strongly with areas that experienced radiologists had marked for additional review, and in 85% of cases, these regions contained clinically significant findings that could have been overlooked without the uncertainty warning system.

#### Quality control and monitoring

Maintaining the reliability of uncertainty quantification systems requires systematic quality control measures [9]. Departments should implement standardized monitoring protocols to track the correlation between uncertainty measures and clinical outcomes [12]. This includes periodic assessment of false positive and false negative rates stratified by uncertainty scores, and regular calibration of uncertainty thresholds based on accumulated clinical data [7]. Documentation of these quality metrics is crucial for both continuous improvement and regulatory compliance. This need for robust quality control and extensive clinical validation data further strengthens the case for establishing centralized imaging hubs, where larger volumes of standardized data and outcomes can be pooled across institutions to enable more reliable uncertainty threshold calibration and comprehensive quality metrics.

## CONCLUSIONS

The continued evolution of deep learning-based uncertainty quantification promises increasingly sophisticated quality assurance capabilities. Integration with other artificial intelligence-driven systems will create more comprehensive solutions for radiological workflow optimization. These advancements influence broader healthcare delivery systems by providing standardized quality metrics and uncertainty measures, facilitating better communication between providers, and eventually will provide more consistent care delivery.

## References

[1] Zhou LQ , et al. World J Gastroenterol. 2019; 25:672–82. 10.3748/wjg.v25.i6.672. 30783371 PMC6378542

[2] Huang L , et al. Med Image Anal. 2024; 97:103223. 10.1016/j.media.2024.103223. 38861770

[3] Maruyama H , et al. Diagnostics (Basel). 2021; 11:292. 10.3390/diagnostics11020292. 33673229 PMC7918339

[4] Litjens G , et al. Med Image Anal. 2017; 42:60–88. 10.1016/j.media.2017.07.005. 28778026

[5] Stollmayer R , et al. World J Gastroenterol. 2021; 27:5978–88. 10.3748/wjg.v27.i35.5978. 34629814 PMC8475009

[6] Daye D , et al. Radiology. 2022; 305:555–63. 10.1148/radiol.212151. 35916673 PMC9713445

[7] Faghani S , et al. Radiology. 2023; 308:e222217. 10.1148/radiol.222217. 37526541

[8] Schooler GR , et al. Radiology. 2020; 296:493–97. 10.1148/radiol.2020200751. 32602829

[9] Tang C , et al. J Magn Reson Imaging. 2024. [Epub ahead of print]. 10.1002/jmri.29672. 39690114 PMC12063763

[10] Lambert B , et al. arXiv. 2023; arXiv:2308.11969. 10.48550/arXiv.2308.11969.

[11] Del Corso G , et al. Comput Struct Biotechnol J. 2024; 24:603–10. 10.1016/j.csbj.2024.09.021. 39421530 PMC11483457

[12] Huang C , et al. Phys Med Biol. 2023; 68. 10.1088/1361-6560/ad027e. 37820639

[13] Shih SF , et al. Magn Reson Med. 2023; 89:1567–85. 10.1002/mrm.29525. 36426730 PMC9892263

[14] Mennella C , et al. Heliyon. 2024; 10:e26297. 10.1016/j.heliyon.2024.e26297. 38384518 PMC10879008

